# Association of mutations in V3/C3 domain with enhanced sensitivity of HIV-1 clade C primary envelopes to autologous broadly neutralizing plasma antibodies

**DOI:** 10.1186/s12977-016-0273-x

**Published:** 2016-06-15

**Authors:** Suprit Deshpande, Shilpa Patil, Rajesh Kumar, Tripti Shrivastava, Aylur K. Srikrishnan, Kailapuri G. Murugavel, Wayne C. Koff, Bimal K. Chakrabarti, Jayanta Bhattacharya

**Affiliations:** HIV Vaccine Translational Research Laboratory, Translational Health Science and Technology Institute, NCR Biotech Science Cluster, Faridabad, Haryana India; Y R Gaitonde Care, Chennai, India; International AIDS Vaccine Initiative, New York, NY USA

**Keywords:** HIV-1, Clade C, Neutralizing antibodies, Envelope, V3/C3

## Abstract

**Background:**

Broadly neutralizing antibodies to HIV-1 elicited in infected individuals evolves through shifts in their molecular specificities to viral envelope (Env) in the disease course. Recently, we showed that resistance of circulating HIV-1 clade C to the autologous plasma obtained from one Indian elite neutralizer is associated with mutations in V1 loop. In the present study, we examined the genetic attributes associated with exceptional sensitivity of pseudoviruses expressing an *env* gene obtained from the follow up visit contemporaneous plasma of the same donor.

**Results:**

Examination of chimeric autologous Envs, we found that enhanced neutralization sensitivity is associated with mutations in the V3/C3 region. A positive association between V3/C3 mutation mediated enhanced autologous neutralization of autologous viruses with their sensitivity to both neutralizing and non-neutralizing monoclonal antibodies was found. Interestingly, we found that depletion of autologous plasma with trimeric and monomeric Envs conferred the sensitive Env with resistance indicating that mutations in V3/C3 region altered Env conformation towards optimal exposure of epitopes targeted by the neutralizing and non-neutralizing antibodies.

**Conclusion:**

In summary, we found distinct vulnerabilities associated with evasion of circulating viruses to broadly neutralizing antibodies mounted in an Indian elite neutralizer.

## Background

Vulnerabilities associated with shift in molecular specificities of the broadly neutralizing antibodies elicited in a small proportion of the Human Immunodeficiency Virus Type 1 (HIV-1) infected individuals (also known as elite neutralizers) in natural infection provide vital information of host-virus interaction towards developmental pathway of protective antibodies [1–4]. The HIV-1 uses its surface trimeric envelope (Env) protein composed of three heterodimers of gp120 and gp41 subunits attached non-covalently for viral entry via interaction with CD4 receptor present on T cells. These Env glycoproteins are heavily glycosylated and are targets of the neutralizing antibodies. Although HIV-1 infected individuals typically develop type-specific autologous neutralizing antibodies [5–7], however rarely few of them move on to develop cross-neutralizing antibodies [8–12]. It is believed that a better understanding of immune evasion mechanisms and events leading to development of broadly neutralizing antibodies in natural infection would rationally inform effective Env based immunogen design that can elicit protective antibodies upon vaccination [3].

Recently in an IAVI Protocol G study, by examining Envs obtained from broadly cross neutralizing (BCN) plasma of an elite neutralizer (G37080) collected at two different time points and spaced between 8 months, we showed that mutations in the V1 loop was associated with autologous neutralization escape [13]. In the present study, we examined an HIV-1 clade C Env (HVTR-PG80v2.eJ7) obtained from the follow up plasma of the same donor (G37080) that significantly differed in its sensitivity to the contemporaneous autologous plasma antibodies compared to the two contemporaneous resistant Envs (HVTR-PG80v2.eJ38 and HVTR-PG80v2.eJ41). The enhanced sensitivity of pseudoviruses expressing HVTR-PG80v2.eJ7 Env to the contemporaneous autologous broadly neutralizing plasma antibodies was found to be associated with mutations in the V3/C3 region and exposure of discontinuous epitopes targeted by neutralizing antibodies with multiple specificities.

## Methods

### Ethics statement

The blood samples were collected under the IAVI Protocol G study as described before [13] following approval and clearance from the Y R Gaitonde Care, Chennai Institutional Review Board (IRB) and the Ethics Committee. The informed consents were obtained from the participating donors to use the samples for assessing neutralizing antibody responses and publishing data keeping anonymity of every donor. The serum and plasma samples collected were shipped to the HIV Vaccine Translational Research Laboratory, Translational Health Science and Technology Institute for further assessment and research on the neutralizing antibody response.

### Plasmids, viruses, antibodies, proteins and cells

Plasmids encoding patient (G37080) derived HIV-1 envelopes representing distinct clades were described previously [13]. TZM-bl cells and monoclonal antibodies were obtained from the NIH AIDS Research Reagents Reference program and from the IAVI Neutralizing Antibody Consortium (NAC). 293T cells were obtained from the American Type Culture Collection (ATCC). Purified BG505-SOSIP.664-D7324 protein was kindly provided by Prof John Moore, Weill Cornell Medical College, New York.

### Depletion of plasma antibodies by monomeric, trimeric Env proteins and MPER peptide

The depletion of the G37080 BCN plasma with soluble Env proteins and MPER peptide was done as described previously [13]. Briefly, 30 mg of beads were used to couple with 1 mg of both monomeric (4-2.J41) and trimeric Env (BG505-SOSIP.664) proteins and 0.5 mg of MPER peptide (C1C) in coupling buffer (0.1M NaBO_4_, 1M (NH_4_)_2_SO_4_; pH 9.4) overnight at 37 °C for 16–24 h. Beads bound to Env proteins were next incubated with blocking buffer [PBS (pH 7.4), 0.1 % bovine serum albumin (BSA; Sigma) and 0.05 % Tween 20] at 37 °C to block the unbound sites. For depletion studies, G37080 visit 2 plasma was diluted to 1:50 in DMEM containing 10 % Fetal Bovine Sera (FBS) and 500 µl of diluted plasma was incubated with 20 µl of beads at room temperature. Unbound plasma antibodies were separated from ones those are bound to protein coated beads using a DynaMag™ 15 magnet as described previously [13]. This step was repeated 4–5 times for depletion of plasma antibodies by monomeric gp120 and 10–12 times in case of BG505-SOSIP.664 coated beads respectively.

### Neutralization assay

Neutralization assays were carried out using TZM-bl cells as described before [13]. Env-pseudotyped viruses were incubated with varying dilutions of depleted plasma antibodies and incubated for an hour at 37 °C CO_2_ incubator under humidified condition and subsequently 1 × 10^4^ TZM-bl cells were added into the mixture in presence of 25 μg/ml DEAE-dextran (Sigma, Inc.).The plates were further incubated for 48 h and the degree of virus neutralization was assessed by measuring relative luminescence units (RLU) in a Luminometer (Victor X2, PerkinElmer Inc.). All the neutralization assays were done in duplicate and repeated at least two times. The effect of point substitutions on neutralization of Env-pseudotyped viruses (as shown in Table 2) were done in triplicates and was repeated three times. A fold change in neutralization titer (ID_50_) (due to amino acid substitution/s) that is equal to or greater than twofold (≥twofold) in our study was considered positive.

### PCR amplification, cloning and mutagenesis of autologous env

Autologous *env* clones were obtained from G37080 plasma by limited dilution PCR with slight modification to the methodology described previously [14]. Briefly, viral RNA were extracted using High Pure viral RNA kit (Roche Inc.) following manufacturer’s protocol and cDNA prepared by RT-PCR using Superscript-III first strand synthesis kit (Invitrogen Inc.). *rev*-*gp160env* genes were amplified from the maximally diluted plasma sample using a Phusion hi fidelity DNA polymerase (New England Biolabs Inc.). The complete *env gene* was purified and ligated into pcDNA 3.1/V5-His-TOPO (Invitrogen Inc.) vector. Chimeric Envs were prepared (Fig. 2a) by overlapping PCR and point substitutions were made by Quikchange II kit (Agilent technologies Inc.) following manufacturer’s protocol and as described previously [13].

### Preparation of Env-pseudotyped viruses

Pseudotyped viruses were prepared by co-transfection of envelope expressing plasmid with env-deleted HIV-1 backbone plasmid (pSG3ΔEnv) into 293T cells in 6-well tissue culture plates using FuGENE6 Transfection Reagent (Promega Inc). Cell supernatants containing pseudotyped viruses were used subsequently in neutralization assays in TZM-bl cells. The reduction of infection of TZM-bl cells by the Env-pseudotyped viruses were determined by measuring luciferase activity using Britelite luciferase substrate (Perkin Elmer) with a Victor X2 Luminometer (Perkin Elmer).

## Results and discussion

### An HIV-1 clade C Env obtained from broadly neutralizing plasma showed exceptional degree of sensitivity to contemporaneous and retrospective autologous plasma antibodies

**Fig. 1:**
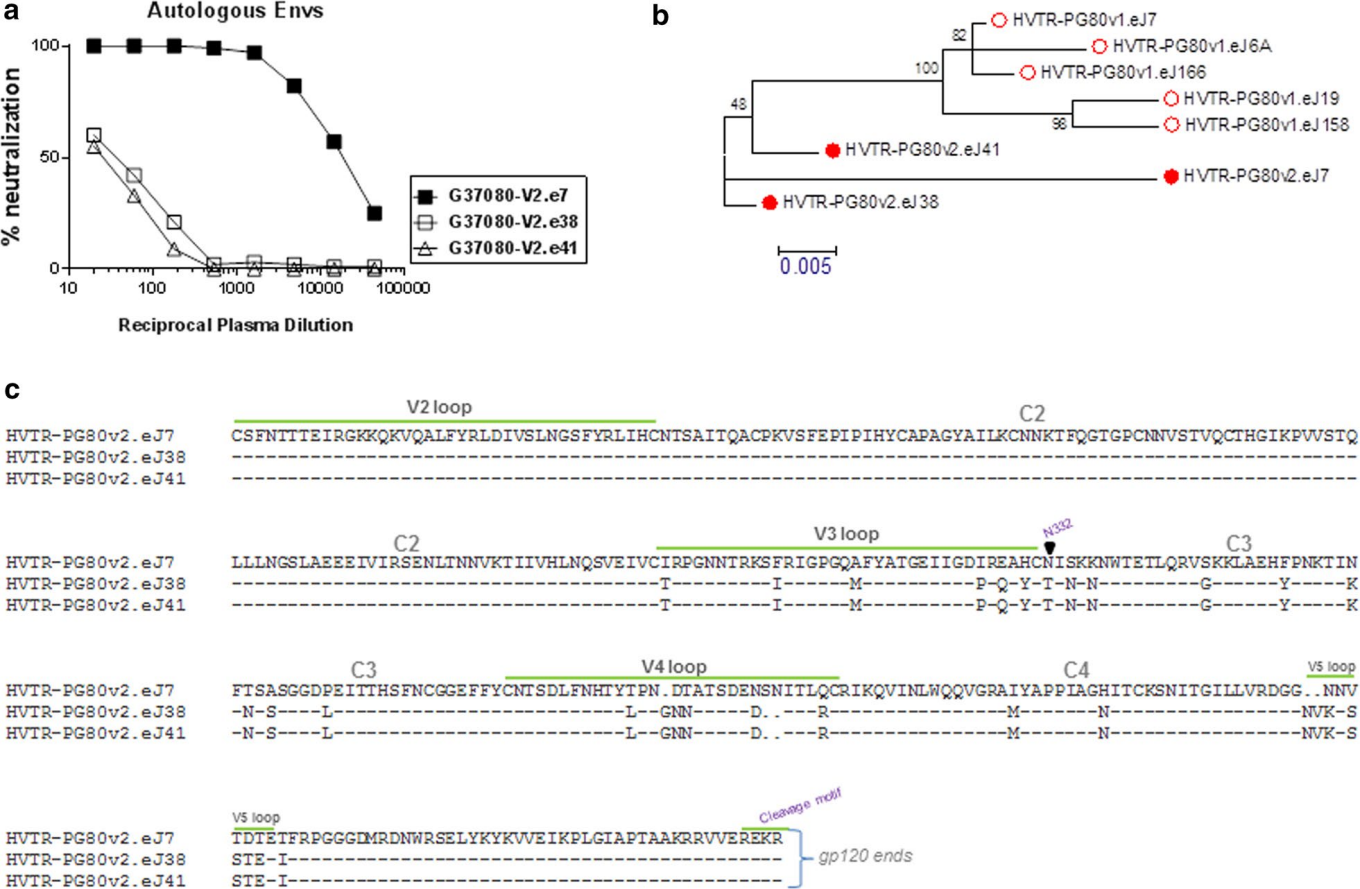
**a** Neutralization of HIV-1 Envs to contemporaneous autologous G37080 BCN plasma. **b** Genetic relatedness of autologous Envs obtained from the plasma of the G37080 donor at two different time points. Maximum likelihood phylogenetic tree was constructed using the amino acid sequences of the viral Envs using Mega 5.1 version. **c** Alignment of the V3–C4 amino acid sequences of the contemporaneous Envs obtained at the same time point

We recently reported [13] pseudoviruses expressing *env* genes obtained from follow up plasma of an Indian elite neutralizer (G37080) those were found to be resistant to their contemporaneous autologous plasma antibodies. We subsequently amplified a functional *env (gp160)* gene (HVTR-PG80v2.eJ7) by PCR from the same plasma, which when expressed as Env-pseudotyped virus showed exceptional sensitivity to its contemporaneous autologous plasma antibodies in sharp contrast to its contemporaneous autologous Envs (HVTR-PG80v2.eJ38 and HVTR-PG80v2.eJ41) (Fig. 1a). Additionally, HVTR-PG80v2.eJ7 unlike HVTR-PG80v2.eJ38 and HVTR-PG80v2.eJ41 Envs was also found to be sensitive to plasma antibodies collected at prior time point (data not shown). Analysis of *gp160* sequence revealed that PG80v2.eJ7 is an HIV-1 clade C Env (http://www.bioafrica.net/rega-genotype/html/) and found to cluster with contemporaneous Envs revealing close genetic relatedness compared to Envs obtained at previous time point (Fig. 1b). Comparison of amino acid sequences revealed that except for intermittent differences in the V3–C4 and V5 regions, HVTR-PG80v2.eJ7 Env was found to be genetically identical to the other two contemporaneous autologous Envs (HVTR-PG80v2.eJ38 and HVTR-PG80v2.eJ41) (Fig. 1c). Interestingly, when compared with all the autologous *env* sequences obtained from both visit 1 and visit 2, HVTR-PG80v2.eJ7 showed >97 % similarity in its amino acid composition (Table 1), indicating that in addition of this Env having conserved structure and function with that of other autologous Envs, it is also clonally and closely related to them (as shown in Fig. 1b), which possess unique property associated with its enhanced susceptibility to autologous BCN plasma antibodies. Overall, we identified an HIV-1 clade C Env obtained from plasma with exceptional breadth which displayed exceptional sensitivity to its contemporaneous autologous plasma, a property which is atypical of circulating viruses in presence of strong humoral immune response.

**Table 1:** Similarity of amino acid sequence of PG80 v2.eJ7 *env* with that of other autologous *envs*

Donor visit #	Autologous *envs*	% Identity^a^	% Similarity^b^
Visit # 2	*PG80v2.eJ7*	*100.0*	*100.0*
	PG80v2.eJ38	95.7	98.0
	PG80v2.eJ41	95.3	98.0
Visit # 1	PG80v1.eJ6a	94.0	97.2
	PG80v1.eJ7	94.3	97.6
	PG80v1.eJ19	92.9	97.3
	PG80v1.eJ158	93.2	97.2
	PG80v1.eJ166	93.8	97.4

LALIGN tool (http://www.ch.embnet.org/software/LALIGN_form.html) was used to obtain sequence identity and similarity

^a^‘% identity’ refers to the degree of correlation between two un-gapped sequences and indicates that the amino acid at the particular position is an exact match

^b^‘ % similarity’ refers to the degree of resemblance between two sequences and indicates that the amino acids at a particular position have some properties in common (e.g., charge or hydrophobicity) but are not identical

### V3/C3 region conferred resistant autologous Env with enhanced sensitivity to contemporaneous plasma antibodies

**Fig. 2:**
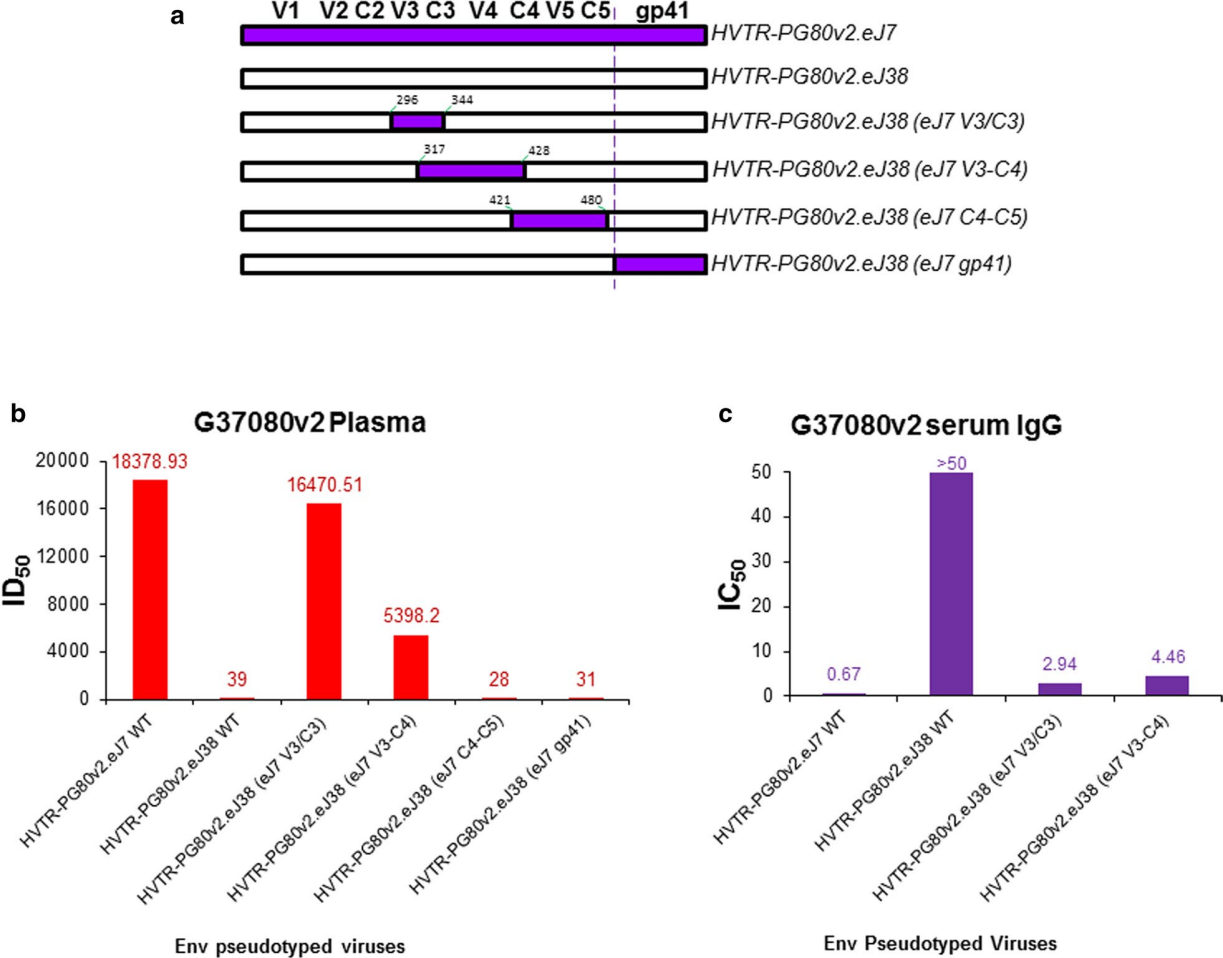
**a** Construction of chimeric Envs using HVTR-PG80v2.eJ38 as backbone. The positions between which the fragments of the HVTR-PG80v2.eJ7 Env were substituted were *highlighted*. The degree of neutralization sensitivities of the wild types and the chimeric Envs to the contemporaneous autologous plasma (**b**) and serum IgG (**c**) are shown

**Fig. 3:**
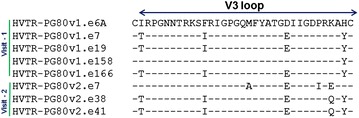
V3 loop amino acid alignment of the autologous *envs*

To examine whether differences in the amino acid sequences accounts for resistance of autologous Envs to the contemporaneous autologous plasma antibodies, we prepared chimeric Envs by replacing V3/C3, V3–C4, C4–C5 and gp41 regions of the resistant Env (HVTR-PG80v2.eJ38) with that of the sensitive Env (HVTR-PG80v2.eJ7) (Fig. 2a). Pseudoviruses expressing chimeric Envs were then examined for their degree of susceptibility to the contemporaneous autologous plasma antibodies. As shown in Fig. 2b, both V3/C3 and V3–C4 chimeras conferred the HVTR-PG80v2.eJ38 Env with enhanced sensitivity by >400 and >130-folds respectively to the contemporaneous autologous plasma. Comparable data was found when serum IgG was tested (Fig. 2c). Interestingly, a difference in V5 sequence between the Envs (Fig. 1c) was not found to be associated with neutralization susceptibility. Our data suggested that residues incorporated from the sensitive HVTR-PG80v2.eJ7 Env altered conformation of the HVTR-PG80v2.eJ38 Env that facilitated autologous plasma antibodies towards comprehensive virus neutralization. Although, we do not rule out the possibility that the plasma antibodies targeted the amino acid residues in the V3 region of the sensitive chimeric envelope; however, when compared, minimal changes between the V3 amino acid sequences of the autologous Envs obtained from both the visits (Fig. 3). The enhanced sensitivity of the v2.eJ38 (v2.eJ7 V3/C3) envelope similar to tier 1 viruses (which in its wild type was found to be resistant to autologous neutralization) due to the V3/C3 residues brought from its contemporaneous envelope possibly form epitopes targeted by the plasma antibodies. Comparable data were obtained when the chimeric viruses were tested against the contemporaneous serum IgG (Fig. 2b), suggesting the neutralization that we observed was indeed antibody mediated. Since substitution of amino acid residues in the V3/C3 domain conferred HVTR-PG80v2.eJ38 Env with maximal sensitivity to the autologous BCN plasma, we further examined whether specific residues within this region significantly modulated the neutralization susceptibility of the HVTR-PG80v2.eJ38 by fine mapping. As shown in Table 2, only P326I substitution in the V3 loop was found to show >eightfold increase in neutralization of HVTR-PG80v2.eJ38, while none other amino acid residues that differed between the sensitive HVTR-PG80v2.eJ7 and the resistant HVTR-PG80v2.eJ38 and HVTR-PG80v2.eJ41 Envs were found to contribute in modulating virus neutralization. Although P326I substitution conferred the HVTR-PG80v2.eJ38 Env with increase in its autologous neutralization susceptibility by >eightfold, it partially restored its neutralization sensitivity. Our data indicates that although P326I had a major effect, presence of other residues in the V3–C4 region in addition to I326 were associated towards achieving comparable neutralization susceptibility of chimeric Envs to that of the wild type. Interestingly, except HVTR-PG80v2.eJ7, none of the autologous Envs obtained from the G37080 donor at two different time points were found to contain the N332 glycan [13]. Additionally, N332T substitution (Table 2) in the v2.eJ7 Env did not alter their susceptibilities to the contemporaneous autologous antibodies, indicating that autologous plasma do not have antibodies that target this glycan epitope. We further examined whether presence of the glycan residues N360 in v2.eJ7 and N362 in v2.eJ38 Envs in the C3 region were associated with enhanced neutralization sensitivity and resistance respectively as these residues were uniquely present in these two Envs only and not to other autologous Envs obtained from visit 1 plasma of the G37080 donor. As shown in Table 2, we did not find any association of these glycan residues with altered susceptibility of the Envs to autologous plasma antibodies. Interestingly, we further observed that the HVTR-PG80v2.eJ38 chimeric Env (V3/C3 chimera) became resistant to the autologous G37080 BCN plasma depleted with BG505-SOSIP.664 soluble trimeric protein (Table 3), indicating that the shift in the neutralization susceptibility of the resistant PG80v2.eJ38 Env to its autologous plasma was associated with exposure of epitopes targeted by both neutralizing antibodies and non-neutralizing antibodies.

**Table 2:** Mapping amino acid residues in the V3/C3 region associated with neutralization sensitivity

Env-pseudotyped viruses (Env chimeras & point mutants)	Fold changes in ID_50_	Effect
HVTR-PG80v2.eJ7 wild type	–	–
HVTR-PG80v2.eJ38 wild type	–	–
HVTR-PG80v2.eJ38(v2.eJ7V3/C3)	>422	Increase
HVTR-PG80v2.eJ38(v2.eJ7V3-C4)	>138	Increase
HVTR-PG80v2.eJ38(v2.eJ7gp41)	0.8	No change
HVTR-PG80v2.eJ38(I307F)	1	No change
HVTR-PG80v2.eJ38(M316A)	1	No change
HVTR-PG80v2.eJ38(P326I)	>8	Increase
HVTR-PG80v2.eJ38(*N*362T)	1	No change
HVTR-PG80v2.eJ7(*N*332T)	1.2	No change
HVTR-PG80v2.eJ7(E328Q)	1.8	Decrease
HVTR-PG80v2.eJ7(T362*N*/A364S)*	1.0	No change

* The T362N/A364S double mutations removed the N360 glycan and introduced N362 glycan in PG80v2.eJ7 Env

Potential N-linked glycans are given in *italic*

**Table 3:** Effect of depletion of G37080 BCN plasma antibodies on autologous virus neutralization

G37080 depleted plasma (fold reduction in ID_50_)				
Env-pseudotyped viruses	gp140 Trimer (BG505-SOSIP.664)	gp120 Monomer (4-2.J41)	MPER (C1C peptide)	References
HVTR-PG80v2.eJ7	>150.0	80.0	1.00	This study
HVTR-PG80v2.eJ38 (v2.eJ7V3/C3)	>70.0	>10.0	1.10	This study
HVTR-PG80v1.eJ7	>10.03	0.9	1.12	[13]
HVTR-PG80v1.eJ19	>15.60	0.5	1.18	[13]
92BR020	>35.08	1.1	1.34	[13]

Fold reduction in virus neutralization was obtained by comparing the neutralization titer (ID_50_ values) of Env-pseudotyped viruses against undepleted and depleted G37080 plasma. ID_50_ values are reciprocal dilutions at which the undepleted and depleted plasma conferred 50 % neutralization of the Env-pseudotyped viruses in TZM-bl cells. ‘v1’ and ‘v2’ refers to Envs obtained from visit 1 and visit 2 plasma samples

### Changes in V3/C3 sequence conferred autologous Envs vulnerable to neutralizing and non-neutralizing mAbs

We next examined the association of neutralization sensitivity of the autologous Envs including the chimeric Envs with their degree of sensitivities to monoclonal antibodies with distinct targets. As shown in Table 4, HVTR-PG80v2.eJ7 wild type Env was found to be sensitive to broadly neutralizing (such as VRC01, PG9, PGT128, PGT135, 10E8), those targeting V3 loop with limited breadth (such as 3074 and 3869), non-neutralizing (b6, F105) and coreceptor binding site (CoRbs) (17b) directed mAbs. On the other hand, the HVTR-PG80v2.eJ38 wildtype was found to be resistant to most of the neutralizing, non-neutralizing and CoRbs directed mAbs. However, fragments of the HVTR-PG80v2.eJ7 Env displayed similar neutralization sensitivity to all the neutralizing and non-neutralizing monoclonal antibodies, suggesting that V3–C4 domain was responsible for exposure of discontinuous epitopes that are targets of neutralizing and non-neutralizing antibodies. HVTR-PG80v2.eJ7 is the only autologous Env which was found to contain N332 in the V3 base compared to all other autologous Envs that contains T332 and hence, compared to its wild type, the HVTR-PG80v2.eJ38 Env containing V3/C3 and V3–C4 regions of the HVTR-PG80v2.eJ7 Env were found to restore its sensitivity to PGT121. Interestingly, while HVTR-PG80v2.eJ38 (V3/C3 chimera) despite expressing N332 was found to be resistant to the PGT128 and PGT135 mAbs, HVTR-PG80v2.eJ38 Env (expressing V3–C4 of HVTR-PG80v2.eJ7) conferred the HVTR-PG80v2.eJ38 Env susceptible to these two mAbs, indicating residues within V3–C4 of the sensitive HVTR-PG80v2.eJ7 Env modulated the conformation of the resistant HVTR-PG80v2.eJ38 Env for optimal exposure of epitopes targeted by PGT128 and PGT135 for comprehensive virus neutralization. The CATNAP database (HIV Los Alamos database; www.hiv.lanl.gov) provides information on the residues that form epitopes for several broad and potent monoclonal antibodies to HIV-1. Accordingly, the following residues are important for PGT128/PGT135 mAbs: N301/T303/R304/I323/G324/N325/M326/R327/N332. We found that both the HVTR-PG80v2.eJ7 (sensitive to autologous neutralization) and the HVTR-PG80v2.eJ38 (resistant to autologous neutralization) contains all these residues with following exceptions: (1) PG80v2.eJ38 lacks N332 residue as pointed above), (2) N325 is absent in both the PG80v2.eJ7 and PG80v2.eJ38 envelopes; both contains D325 (aspartic acid) and (3) M326 is absent in both the envelopes; while PG80v2.eJ7 contains I326 (an isoleucine residue), the PG80v2.eJ38 envelope contains P326 (a proline residue). Interestingly, neither the PG80v2.eJ38 envelope nor its chimera (expressing the V3/C3 domain of the PG80v2.eJ7 envelope) was found to show modest resistance to the PGT128/PGT135 mAbs. On the other hand, the PG80v2.eJ38 chimeric envelope expressing V3–C4 domain of the PG80v2.eJ7 envelope was found to show sensitivity to both PGT128 and PGT135 mAbs, suggesting that while V3/C3 swap conferred the resistant PG80v2.eJ38 envelope with exposure of epitopes that are targeted by the non-neutralizing antibodies e.g., b6, 17b, V3 directed mAbs (3074 and 3869) including those present in the autologous plasma; however the V3–C4 swap enabled optimal exposure of neutralizing epitopes targeted by potent and broadly neutralizing antibodies such as PGT128 and PGT135. Overall, our data indicate that subtle changes in V3–C4 region is capable of concealing key epitopes associated with broad neutralization which recognizes PGT128 and PGT135 like mAbs by altering Env conformations.

**Table 4:**
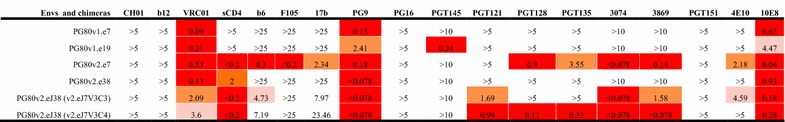
Sensitivity of autologous Envs to mAbs

Values are mAb doses (µg/ml) at which 50 % virus neutralization are observed in TZM-bl cells. IC_50_ values less than 1, 3 and 5 µg/ml are highlighted in red, orange and light pink respectively

### Association of presence of D167 in V2 apex with resistance to the PGT145 mAb

**Fig. 4:**
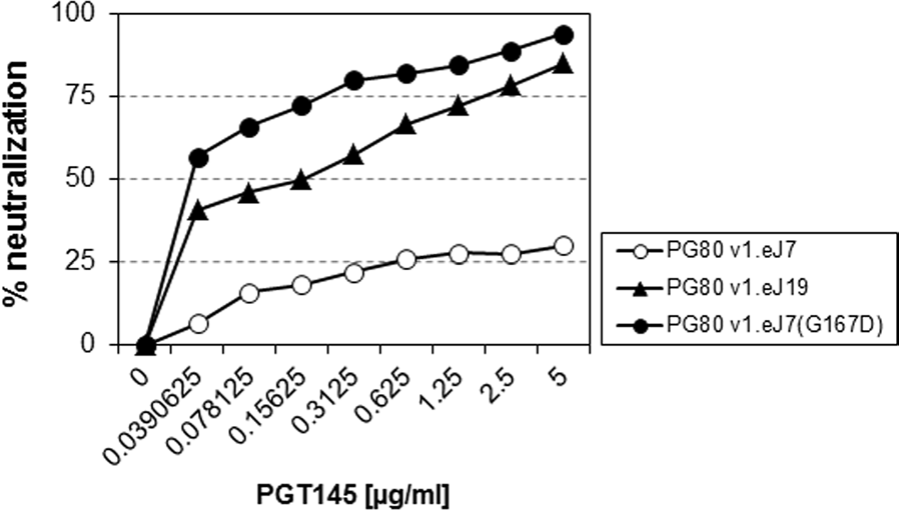
Effect of G167D mutation on sensitivity of Env-pseudotyped viruses to the PGT145 mAb

Additionally, we found that except for HVTR-PG80v1.eJ19 (obtained from visit 1 plasma) [13], all the autologous Envs obtained from both visit plasma were resistant to PGT145 mAb. Interestingly, we found that the presence of G167 residue in all the autologous Envs (except the HVTR-PG80v2.eJ19 Env, which was found to possess D167) was found to be correlated with their resistance to PGT145 mAb. A G167D substitution was found to confer the autologous Envs sensitive to PGT145 mAb with IC_50_ value of <0.04 µg/ml (Fig. 4). This is an interesting observation since G167 amino acid residue forms one of the epitopes for PGT145 mAb recognition in majority of the HIV-1 strains (www.hiv.lanl.gov) and no association of mutation of this residue with PGT145 resistance was reported. Our data contribute information to the repertoire of knowledge of mechanism modulating the sensitivity of HIV-1 to the cleavage-dependent bnAb, PGT145.

## Conclusion

In summary, we report an HIV-1 clade C Env (HVTR-PG80v2.eJ7) from the G37080 BCN plasma of an elite neutralizer of Indian origin which displayed exceptional sensitivity to its contemporaneous autologous plasma antibodies. The HVTR-PG80v2.eJ38 resistant Env expressing the V3/C3 sequence of the HVTR-PG80v2.eJ7 Env was found to accommodate conformational epitopes targeted by the neutralizing antibodies present in the G37080 plasma as evident by the BCN plasma depletion study. Overall, we found that initially while the viruses circulating early escaped from the broadly neutralizing autologous antibodies via mutations in V1 loop that we recently reported [13], they subsequently evaded the antibody pressure in the disease course by accommodating mutations in V3/C3 region. Understanding pathways of co-evolution of circulating virus and neutralizing antibodies specific to them will complement strategies of rational immunogen design towards achieving elicitation of antibodies with similar breadth and potency.
